# Trends in Prevalence of Adverse Childhood Experiences Among Children

**DOI:** 10.1001/jamanetworkopen.2025.44425

**Published:** 2025-11-19

**Authors:** Guodong Ding, Anqi Peng, Yan Chen, Angela Vinturache, Yongjun Zhang

**Affiliations:** 1 Department of Pediatrics, Xinhua Hospital, Shanghai Jiao Tong University School of Medicine, Shanghai, China; 2 Clinical Medical College, Yangzhou University, Yangzhou, Jiangsu Province, China; 3 Department of Obstetrics & Gynecology, University of Alberta, Edmonton, Alberta, Canada

## Abstract

This cross-sectional study uses data from the National Survey of Children’s Health to analyze temporal changes in the prevalence of 0 vs 4 or more adverse childhood experiences among US children.

## Introduction

Adverse childhood experiences (ACEs), which are preventable and potentially traumatic events that occur in childhood, include harms that affect children directly and indirectly through their living environments.^1^ ACEs are consistently linked to adverse behavioral, health, and social outcomes across the lifespan, with individuals having 4 or more ACEs at greater risk for all health consequences compared with those with none.^2^ From 2017 to 2018, among US children aged 0 to 17 years, the prevalence rate of 0 ACEs was 58.5%, and that of 4 or more ACEs was 5.3%.^3^ This study used the latest national data to analyze temporal changes in the prevalence of 0 and 4 or more ACEs among US children.

## Methods

The US National Survey of Children’s Health (NSCH) annually collects data from parents or primary caregivers via mail or web questionnaires to monitor the health and well-being of noninstitutionalized children aged 0 to 17 years.^4^ This cross-sectional study followed the STROBE reporting guideline. As the data used were publicly available and anonymized, the study was deemed exempt from institutional review board oversight in accordance with 45 CFR §46. Nine ACEs were assessed in the NSCH: economic hardship, divorce, death of a parent and/or guardian, history of incarceration of a parent and/or guardian, domestic violence exposure, neighborhood violence exposure, mental illness of a parent and/or guardian living within the household, alcohol and/or drug problem of a parent and/or guardian living within the household, and racial discrimination. We summed the responses across all 9 binary items (yes or no) to create a measure indicating whether the child experienced no ACEs or 4 or more ACEs, respectively. We analyzed the NSCH data from 2016 to 2023 to evaluate trends in the prevalence of 0 and 4 or more ACEs among US children aged 0 to 17 years, both overall and by sociodemographic subgroups, using a survey-weighted logistic regression model, with the survey year treated as a continuous variable. Child’s sex, race and ethnicity, age group, family income, highest education of parent and/or guardian, and family size were mutually adjusted as appropriate.^5^ All analyses were performed using R statistical software version 4.4.1 (R Project for Statistical Computing). Two-tailed *P* < .05 indicates statistical significance.

## Results

Among 334 708 participants, 21 742 were excluded due to missing ACEs data, leaving 312 966 (161 701 boys [51.2%]) for this study. In 2023, 37.7% (95% CI, 36.8%-38.6%) of children had at least 1 ACE. The overall prevalence of 0 ACEs was 59.8% (95% CI, 59.4%-60.2%), an increase from 54.5% (95% CI, 53.4%-55.5%) in 2016 to 62.3% (95% CI, 61.4%-63.2%) in 2023, reflecting an 14.3% relative increase (*P* < .001 for trend) (Table 1). Stratification across all sociodemographic subgroups showed trends similar to the overall population.

**Table 1.** Trends in Prevalence of 0 ACEs Among US Children Aged 0 to 17 Years From the National Survey of Children’s Health, 2016 to 2023^a^

ACEs^b^	Children, No. (%) [95% CI]									Absolute (relative) difference, 2023 vs 2016, %^c^	*P *value for trend^d^
	2016	2017	2018	2019	2020	2021	2022	2023	Overall		
Total	27 883 (54.5) [53.4-55.5]	12 042 (56.5) [55.0-58.0]	17 826 (60.4) [59.2-61.6]	17 030 (60.4) [59.1-61.6]	25 230 (60.8) [59.8-61.9]	31 198 (62.6) [61.6-63.6]	32 563 (60.9) [60.0-61.8]	34 111 (62.3) [61.4-63.2]	197 883 (59.8) [59.4-60.2]	7.8 (14.3)	<.001
Child sex^e^											
Girls	13 564 (55.1) [53.5-56.6]	5876 (57.4) [55.3-59.4]	8513 (59.9) [58.1-61.6]	8101 (59.1) [57.3-60.9]	12 280 (60.5) [59.0-62.0]	15 027 (62.8) [61.4-64.2]	15 781 (61.2) [59.9-62.4]	16 687 (62.6) [61.3-63.8]	95 829 (59.8) [59.2-60.3]	7.5 (13.7)	<.001
Boys	14 319 (54.0) [52.5-55.4]	6166 (55.7) [53.6-57.7]	9313 (60.9) [59.2-62.5]	8929 (61.6) [59.9-63.3]	12 950 (61.2) [59.7-62.6]	16 171 (62.4) [61.0-63.8]	16 782 (60.6) [59.4-61.9]	17 424 (62.0) [60.8-63.3]	102 054 (59.8) [59.2-60.3]	8.1 (15.0)	<.001
Race and ethnicity^f^											
Hispanic	2556 (49.7) [46.7-52.6]	1180 (53.7) [49.6-57.9]	1855 (58.9) [55.5-62.2]	1805 (58.2) [54.8-61.6]	2936 (55.9) [53.2-58.7]	3757 (60.3) [57.6-62.9]	4358 (57.3) [55.2-59.5]	4310 (58.1) [56.0-60.3]	22 757 (56.6) [55.5-57.6]	8.5 (17.0)	<.001
Non-Hispanic Black	1073 (37.5) [34.4-40.6]	472 (38.5) [34.0-43.1]	807 (47.2) [43.5-51.0]	746 (44.8) [40.8-48.8]	1158 (45.8) [42.6-49.0]	1364 (47.4) [44.3-50.5]	1280 (42.7) [39.6-45.7]	1421 (48.4) [45.4-51.4]	8321 (44.0) [42.7-45.2]	11.0 (29.2)	<.001
Non-Hispanic White	20 880 (60.0) [59.0-61.0]	8861 (61.3) [59.9-62.8]	13 051 (63.4) [62.2-64.6]	12 460 (64.5) [63.3-65.7]	17 695 (65.6) [64.5-66.8]	21 775 (67.2) [66.1-68.2]	22 213 (66.2) [65.2-67.1]	23 354 (66.9) [65.9-67.9]	140 289 (64.3) [63.9-64.7]	6.9 (11.5)	<.001
Other	3374 (59.8) [57.1-62.6]	1529 (61.1) [57.3-64.9]	2113 (64.9) [61.8-68.1]	2019 (64.1) [61.1-67.0]	3441 (67.1) [64.6-69.6]	4302 (64.2) [61.8-66.7]	4712 (64.7) [62.7-66.7]	5026 (66.8) [64.9-68.7]	26 516 (64.2) [63.3-65.2]	7.0 (11.6)	.02
Age, y											
0-5	9797 (66.1) [64.2-67.9]	4172 (66.4) [63.7-69.1]	6281 (73.9) [71.8-76.0]	5904 (73.8) [71.8-75.8]	9022 (74.4) [72.6-76.2]	15 310 (75.3) [73.8-76.7]	14 274 (73.8) [72.4-75.2]	15 868 (75.2) [73.9-76.6]	80 628 (72.3) [71.6-73.0]	9.2 (13.9)	<.001
6-12	9663 (52.2) [50.5-53.9]	4211 (55.3) [53.0-57.6]	6218 (57.8) [55.9-59.7]	6030 (58.4) [56.3-60.4]	8758 (58.1) [56.4-59.8]	9453 (61.9) [60.3-63.5]	10 419 (60.4) [58.9-61.8]	10 626 (61.1) [59.7-62.6]	65 378 (58.1) [57.5-58.8]	8.9 (17.1)	<.001
13-17	8423 (44.4) [42.6-46.1]	3659 (46.7) [44.1-49.3]	5327 (48.5) [46.3-50.7]	5096 (47.9) [45.8-50.0]	7450 (49.1) [47.4-50.9]	6435 (49.3) [47.3-51.3]	7870 (48.5) [46.9-50.1]	7617 (50.6) [49.0-52.2]	51 877 (48.1) [47.4-48.8]	6.2 (14.0)	.002
Family income, % federal poverty level^g^											
<100	1361 (33.6) [30.9-36.3]	927 (39.5) [35.5-43.4]	1344 (45.0) [41.7-48.3]	1204 (43.5) [40.2-46.9]	2128 (44.7) [41.9-47.6]	2639 (47.0) [44.3-49.7]	2715 (42.2) [40.0-44.5]	2720 (45.5) [43.0-48.0]	15 038 (42.4) [41.4-43.5]	11.9 (35.4)	<.001
100-199	2960 (43.0) [40.6-45.4]	1232 (44.5) [40.9-48.2]	2154 (49.9) [47.0-52.7]	2054 (50.8) [47.9-53.7]	3152 (49.2) [46.8-51.7]	3838 (54.7) [52.5-57.0]	3834 (49.3) [47.3-51.4]	4022 (50.5) [48.4-52.5]	23 245 (48.9) [47.9-49.8]	7.5 (17.4)	<.001
200-399	8243 (56.9) [55.3-58.5]	3409 (56.3) [53.9-58.7]	5285 (61.2) [59.3-63.2]	5231 (60.7) [58.5-62.8]	7534 (61.0) [59.2-62.7]	9343 (61.8) [60.1-63.5]	9175 (60.2) [58.6-61.8]	9762 (62.1) [60.6-63.5]	57 983 (60.1) (59.4-60.7)	5.2 (9.1)	<.001
≥400	15 319 (75.0) [73.8-76.1]	6474 (76.0) [74.4-77.6]	9043 (75.8) [74.3-77.3]	8541 (76.3) [74.8-77.8]	12 416 (77.2) [76.0-78.4]	15 378 (77.0) [75.8-78.2]	16 839 (77.8) [76.8-78.8]	17 607 (77.6) [76.6-78.7]	101 617 (76.6) [76.2-77.1]	2.7 (3.6)	.002
Highest education of parent or guardian^h^											
Less than college degree^i^	7261 (41.4) [39.7-43.1]	3229 (44.1) [41.7-46.6]	5365 (49.5) [47.5-51.5]	4809 (48.7) [46.7-50.8]	7311 (49.4) [47.6-51.1]	8642 (52.0) [50.3-53.7]	8836 (48.3) [46.8-49.7]	8591 (49.6) [48.1-51.1]	54 044 (47.8) [47.1-48.5]	8.2 (19.7)	<.001
College graduate or higher	20 470 (68.4) [67.3-69.5]	8813 (69.0) [67.5-70.6]	12 461 (71.2) [70.0-72.5]	12 221 (71.6) [70.3-72.9]	17 919 (71.5) [70.4-72.6]	22 556 (72.4) [71.3-73.4]	23 727 (71.7) [70.7-72.7]	25 520 (72.7) [71.8-73.7]	143 687 (71.1) [70.7-71.5]	4.3 (6.3)	<.001
Family size^h^											
1-3	8482 (41.3) [39.7-43.0]	3753 (42.2) [39.9-44.6]	5423 (47.1) [45.0-49.1]	4970 (45.5) [43.6-47.5]	7511 (47.4) [45.7-49.1]	8989 (45.5) [43.8-47.2]	9797 (47.6) [46.1-49.1]	10 170 (47.7) [46.2-49.2]	59 095 (45.5) [44.9-46.2]	6.4 (15.4)	<.001
4-5	16 716 (62.0) [60.6-63.3]	7423 (64.4) [62.7-66.2]	11 039 (66.5) [65.0-68.1]	10 721 (67.1) [65.5-68.6]	15 668 (67.9) [66.5-69.2]	19 415 (69.5) [68.3-70.8]	20 001 (67.6) [66.4-68.7]	20 694 (68.8) [67.6-69.9]	121 677 (66.7) [66.2-67.2]	6.8 (11.0)	<.001
≥6	2035 (51.9) [48.4-55.3]	769 (53.2) [47.9-58.5]	1254 (61.8) [57.8-65.9]	1201 (62.5) [58.5-66.4]	1865 (58.5) [55.0-62.0]	2495 (67.4) [64.6-70.3]	2447 (58.2) [55.3-61.0]	2916 (63.6) [61.0-66.2]	14 982 (59.6) [58.3-60.9]	11.7 (22.6)	<.001

Abbreviation: ACE, adverse childhood experience.

^a^ All estimates, except sample sizes, are weighted.

^b^ The 9 ACEs are described in the Methods section.

^c^ Absolute differences in estimated prevalence between 2016 and 2023 were calculated by treating each survey cycle as an indicator category, with the 2016 cycle as the reference. Values may not equal the difference between the beginning and ending estimates because of rounding. Relative difference, presented as a percentage, is the absolute difference divided by the prevalence in the referent category (2016) multiplied by 100.

^d^ Child’s sex, race and ethnicity, age group, family income, highest education of parent or guardian, and family size were mutually adjusted as appropriate.

^e^ Child sex (0.1% missing) was imputed by the Census Bureau using hot-deck imputation.

^f^ Race and ethnicity (child’s Hispanic origin had 0.4% and race had 1.9% missing cases) was imputed by the Census Bureau using hot-deck imputation. Other race includes American Indian or Alaska Native, Asian, Native Hawaiian and Other Pacific Islander, some other race, and 2 or more races.

^g^ Family income (15.9%) was imputed by the Census Bureau using sequential regression imputation methods.

^h^ Numbers vary slightly in the subgroups due to missing data.

^i^ Includes less than high school, high school, and some college or technical school.

The overall prevalence of 4 or more ACEs was 5.2% (95% CI, 5.0%-5.4%), a decrease from 5.9% (95% CI, 5.4%-6.4%) in 2016 to 4.9% (95% CI, 4.5%-5.3%) in 2023, reflecting a 17.4% relative decrease (*P* = .006 for trend) (Table 2). The decreasing trend was more pronounced among other racial and ethnic groups and those from low-income families, with respective decreases of 30.3% and 29.7%.

**Table 2.** Trends in Prevalence of 4 or More ACEs Among US Children Aged 0 to 17 Years From the National Survey of Children’s Health, 2016 to 2023^a^

ACEs^b^	Children, No. (%) [95% CI]									Absolute (relative) difference, 2023 vs 2016, %^c^	*P *value for trend^d^
	2016	2017	2018	2019	2020	2021	2022	2023	Overall		
Total	2306 (5.9) [5.4 to 6.4]	1051 (5.4) [4.7 to 6.0]	1517 (5.6) [5.0 to 6.1]	1423 (5.3) [4.7 to 5.9]	1939 (4.9) [4.5 to 5.4]	2077 (4.6) [4.2 to 5.0]	2236 (5.0) [4.6 to 5.4]	2202 (4.9) [4.5 to 5.3]	14 751 (5.2) [5.0 to 5.4]	−1.0 (−17.4)	.006
Child sex^e^											
Girls	1161 (5.8) [5.1 to 6.5]	525 (5.0) [4.2 to 5.9]	696 (5.9) [5.0 to 6.7]	687 (5.6) [4.7 to 6.5]	924 (4.6) [4.0 to 5.2]	1053 (5.0) [4.4 to 5.7]	1093 (5.2) [4.6 to 5.7]	1059 (4.8) [4.3 to 5.3]	7198 (5.2) [5.0 to 5.5]	−1.0 (−17.7)	.13
Boys	1145 (6.0) [5.3 to 6.7]	526 (5.7) [4.8 to 6.6]	821 (5.3) [4.6 to 6.0]	736 (5.1) [4.3 to 5.9]	1015 (5.2) [4.5 to 5.9]	1024 (4.1) [3.7 to 4.6]	1143 (4.8) [4.2 to 5.4]	1143 (4.9) [4.3 to 5.6]	7553 (5.2) [4.9 to 5.4]	−1.0 (−17.2)	.02
Race and ethnicity^f^											
Hispanic	334 (5.6) [4.5 to 6.7]	147 (4.6) [3.2 to 6.0]	223 (5.3) [3.8 to 6.7]	206 (5.1) [3.6 to 6.6]	317 (4.7) [3.5 to 5.8]	330 (3.7) [2.8 to 4.5]	397 (5.0) [3.9 to 6.0]	404 (4.5) [3.6 to 5.3]	2358 (4.8) [4.4 to 5.2]	−1.2 (−20.8)	.13
Non-Hispanic Black	194 (9.2) [7.0 to 11.4]	113 (9.2) [6.4 to 11.9]	134 (6.9) [5.1 to 8.6]	102 (8.9) [6.2 to 11.6]	194 (8.1) [6.3 to 9.8]	186 (7.1) [5.1 to 9.1]	184 (7.2) [5.6 to 8.7]	190 (7.1) [5.4 to 8.8]	1297 (8.0) [7.2 to 8.7]	−2.1 (−23.0)	.10
Non-Hispanic White	1463 (5.1) [4.6 to 5.6]	648 (4.8) [4.2 to 5.5]	955 (5.1) [4.5 to 5.7]	886 (4.7) [4.1 to 5.2]	1142 (4.1) [3.7 to 4.6]	1253 (4.5) [4.1 to 4.9]	1331 (4.5) [4.0 to 4.9]	1297 (4.6) [4.2 to 5.1]	8975 (4.7) [4.5 to 4.9]	−0.4 (−8.8)	.42
Other	315 (6.3) [4.8 to 7.9]	143 (5.2) [3.8 to 6.6]	205 (7.0) [4.9 to 9.0]	229 (5.0) [3.9 to 6.2]	286 (5.6) [4.2 to 6.9]	308 (4.3) [3.5 to 5.1]	324 (5.0) [4.2 to 5.8]	311 (4.4) [3.5 to 5.4]	2121 (5.3) [4.9 to 5.8]	−1.9 (−30.3)	.045
Age, y											
0-5	251 (2.3) [1.8 to 2.7]	110 (2.0) [1.2 to 2.9]	151 (1.9) [1.3 to 2.4]	144 (2.1) [1.5 to 2.8]	193 (2.3) [1.6 to 3.0]	280 (1.8) [1.4 to 2.3]	291 (1.8) [1.4 to 2.2]	288 (1.6) [1.3 to 1.9]	1708 (2.0) [1.8 to 2.2]	−0.6 (−28.4)	.62
6-12	863 (6.5) [5.6 to 7.4]	406 (5.9) [4.9 to 7.0]	601 (6.2) [5.3 to 7.1]	577 (5.3) [4.4 to 6.1]	772 (5.0) [4.3 to 5.7]	721 (4.6) [3.9 to 5.2]	828 (5.4) [4.7 to 6.2]	792 (5.1) [4.4 to 5.8]	5560 (5.5) [5.2 to 5.8]	−1.4 (−22.0)	.02
13-17	1192 (9.2) [8.1 to 10.3]	535 (8.4) [7.1 to 9.8]	765 (8.9) [7.5 to 10.3]	702 (9.1) [7.6 to 10.6]	974 (7.8) [6.8 to 8.8]	1076 (7.7) [6.6 to 8.7]	1117 (7.6) [6.7 to 8.5]	1122 (7.9) [7.0 to 8.8]	7483 (8.3) [7.9 to 8.7]	−1.3 (−14.0)	.11
Family income, % federal poverty level^g^											
<100	608 (11.5) [10.0 to 13.1]	268 (8.3) [6.4 to 10.2]	399 (11.2) [9.1 to 13.2]	334 (9.7) [7.8 to 11.5]	532 (9.1) [7.4 to 10.7]	571 (8.1) [6.6 to 9.6]	608 (9.6) [8.1 to 11.1]	582 (8.1) [7.1 to 9.1]	3902 (9.5) [8.9 to 10.1]	−3.4 (−29.7)	.01
100-199	667 (7.5) [6.4 to 8.6]	284 (8.3) [6.6 to 10.1]	443 (7.8) [6.5 to 9.2]	436 (8.6) [6.9 to 10.4]	559 (7.4) [6.3 to 8.4]	567 (5.8) [5.0 to 6.6]	645 (7.6) [6.5 to 8.6]	649 (8.1) [6.9 to 9.3]	4250 (7.7) [7.2 to 8.1]	0.6 (7.9)	.27
200-399	659 (4.7) [3.9 to 5.5]	333 (4.7) [3.8 to 5.7]	447 (4.3) [3.6 to 5.0]	419 (4.5) [3.5 to 5.4]	569 (4.6) [3.8 to 5.5]	621 (4.7) [4.0 to 5.4]	643 (4.3) [3.6 to 5.0]	657 (4.7) [3.9 to 5.5]	4348 (4.6) [4.3 to 4.8]	0.01 (0.1)	.36
≥400	372 (2.0) [1.5 to 2.4]	166 (2.0) [1.4 to 2.6]	228 (1.9) [1.4 to 2.3]	234 (1.4) [1.1 to 1.8]	279 (1.3) [1.1 to 1.6]	318 (1.7) [1.3 to 2.1]	340 (1.7) [1.4 to 2.1]	314 (1.6) [1.3 to 1.9]	2251 (1.7) [1.6 to 1.8]	−0.4 (−20.7)	.23
Highest education of parent or guardian^h^											
Less than college degree^i^	1489 (8.6) [7.8 to 9.5]	671 (7.7) [6.6 to 8.8]	1011 (8.2) [7.2 to 9.1]	911 (8.5) [7.4 to 9.7]	1312 (7.5) [6.7 to 8.3]	1326 (6.6) [5.8 to 7.3]	1480 (7.8) [7.0 to 8.5]	1425 (7.6) [6.8 to 8.4]	9625 (7.8) [7.5 to 8.1]	−1.0 (−11.7)	.03
College graduate or higher	769 (2.9) [2.4 to 3.3]	380 (3.1) [2.5 to 3.6]	506 (3.0) [2.4 to 3.6]	512 (2.3) [1.9 to 2.6]	627 (2.5) [2.0 to 3.0]	751 (2.7) [2.3 to 3.1]	756 (2.6) [2.2 to 3.0]	777 (2.6) [2.2 to 3.0]	5078 (2.7) [2.5 to 2.9]	−0.3 (−9.1)	.10
Family size^h^											
1-3	1162 (8.4) [7.5 to 9.4]	582 (7.8) [6.6 to 9.0]	787 (7.3) [6.4 to 8.3]	764 (8.2) [6.9 to 9.5]	1051 (6.9) [6.0 to 7.7]	1096 (7.1) [6.3 to 8.0]	1154 (7.1) [6.4 to 7.8]	1149 (7.4) [6.7 to 8.2]	7745 (7.5) [7.2 to 7.9]	−1.0 (−12.2)	.23
4-5	857 (4.6) [4.0 to 5.2]	378 (4.1) [3.4 to 4.9]	591 (4.4) [3.7 to 5.1]	539 (3.8) [3.2 to 4.5]	698 (3.5) [3.0 to 4.0]	774 (3.5) [3.0 to 4.1]	839 (3.9) [3.4 to 4.5]	809 (3.5) [3.1 to 3.9]	5485 (3.9) [3.7 to 4.1]	−1.1 (−23.6)	.03
≥6	215 (6.3) [4.8 to 7.8]	84 (5.8) [3.7 to 8.0]	119 (6.6) [4.5 to 8.6]	108 (6.5) [4.2 to 8.8]	174 (7.0) [5.1 to 8.8]	177 (4.0) [3.0 to 5.1]	213 (5.6) [4.1 to 7.0]	220 (5.2) [3.8 to 6.6]	1310 (5.9) [5.3 to 6.5]	−1.2 (−18.6)	.20

Abbreviation: ACE, adverse childhood experience.

^a^ All estimates, except sample sizes, are weighted.

^b^ The 9 ACEs are described in the Methods section.

^c^ Absolute differences in estimated prevalence between 2016 and 2023 were calculated by treating each survey cycle as an indicator category, with the 2016 cycle as the reference. Values may not equal the difference between the beginning and ending estimates because of rounding. Relative difference, presented as a percentage, is the absolute difference divided by the prevalence in the referent category (2016) multiplied by 100.

^d^ Child’s sex, race and ethnicity, age group, family income, highest education of parent or guardian, and family size were mutually adjusted as appropriate.

^e^ Child sex (0.1% missing) was imputed by the Census Bureau using hot-deck imputation.

^f^ Race and ethnicity (child’s Hispanic origin had 0.4% and race had 1.9% missing cases) was imputed by the Census Bureau using hot-deck imputation. Other race includes American Indian or Alaska Native, Asian, Native Hawaiian and Other Pacific Islander, some other race, and 2 or more races.

^g^ Family income (15.9%) was imputed by the Census Bureau using sequential regression imputation methods.

^h^ Numbers vary slightly in the subgroups due to missing data.

^i^ Includes less than high school, high school, and some college or technical school.

## Discussion

This cross-sectional study reveals an encouraging positive shift in the prevalence of ACEs among US children during the study period. The upward trends in the proportion of individuals reporting 0 ACEs suggest a growing societal recognition of the importance of healthy and nurturing environments for children.^6^ Conversely, the decrease in the prevalence of 4 or more ACEs highlights a reduction in severe ACEs, particularly among other racial and ethnic groups and those from low-income families. Despite these overall improvements, the 37.7% prevalence of at least 1 ACE and 4.9% prevalence of 4 or more ACEs in 2023 warrant attention, as these experiences can lead to substantial long-term health consequences. Study limitations include parent-reported ACE exposures, incomplete questions regarding ACEs, and the omission of positive childhood adverse experiences. Although child age was a covariate, we did not account for bias from differential ACE exposure, as younger children, with shorter lifespans, are less likely to accumulate multiple ACEs, potentially underestimating prevalence. These findings highlight the necessity for ongoing support and interventions to further reduce ACEs, particularly among at-risk groups, ensuring healthier developmental outcomes for all children.

## Notes

Supplement. Data Sharing Statement
