# Supplementary material for: Trends in Prevalence of Adverse Childhood Experiences Among Children
Source: JAMA Netw Open. 2025 Nov 19;8(11):e2544425. doi: 10.1001/jamanetworkopen.2025.44425 (PMC12631492; doi:10.1001/jamanetworkopen.2025.44425)
Supplement: Supplement. — Data Sharing Statement [file jamanetwopen-e2544425-s001.pdf]

# Data Sharing Statement

Ding. Trends in Prevalence of Adverse Childhood Experiences Among Children. *JAMA Netw Open*. Published November 19, 2025. doi:10.1001/jamanetworkopen.2025.44425

**Data**
**Data available:** Yes
**Data types:** Deidentified participant data
**How to access data:** Data sharing will be available from YZ upon a reasonable request. Electronic address: zhangyongjun@sjtu.edu.cn.
**When available:** beginning date: 12-01-2025, end date: 12-01-2026

**Supporting Documents**
**Document types:** Statistical/analytic code
**How to access documents:** Data sharing will be available from YZ upon a reasonable request. Electronic address: zhangyongjun@sjtu.edu.cn.
**When available:** beginning date: 12-01-2025, end date: 12-01-2026

**Additional Information**
**Who can access the data:** Data sharing will be available from YZ upon a reasonable request. Electronic address: zhangyongjun@sjtu.edu.cn.
**Types of analyses:** Data sharing will be available from YZ upon a reasonable request. Electronic address: zhangyongjun@sjtu.edu.cn.
**Mechanisms of data availability:** Data sharing will be available from YZ upon a reasonable request. Electronic address: zhangyongjun@sjtu.edu.cn.
